# Bioplastics and Carbon-Based Sustainable Materials, Components, and Devices: Toward Green Electronics

**DOI:** 10.1021/acsami.1c13787

**Published:** 2021-10-05

**Authors:** Éva Bozó, Henri Ervasti, Niina Halonen, Seyed Hossein Hosseini Shokouh, Jarkko Tolvanen, Olli Pitkänen, Topias Järvinen, Petra S. Pálvölgyi, Ákos Szamosvölgyi, András Sápi, Zoltan Konya, Marta Zaccone, Luana Montalbano, Laurens De Brauwer, Rakesh Nair, Vanesa Martínez-Nogués, Leire San Vicente Laurent, Thomas Dietrich, Laura Fernández de Castro, Krisztian Kordas

**Affiliations:** † Microelectronics Research Unit, Faculty of Information Technology and Electrical Engineering, University of Oulu, PO Box 4500, FI-90570 Oulu, Finland; ‡ Department of Applied and Environmental Chemistry, University of Szeged, Rerrich B. tér 1, Szeged 6720, Hungary; § MTA-SZTE Reaction Kinetics and Surface Chemistry Research Group, University of Szeged, Rerrich B. tér 1, Szeged 6720, Hungary; ∥ Proplast—Consorzio per la Promozione della Cultura Plastica, Via Roberto di Ferro, 86, 15122 Alessandria (AL), Italy; ⊥ Bio Base Europe Pilot Plant VZW, Rodenhuizekaai 1, 9042 Desteldonk (Gent), Belgium; # Tecnopackaging, Polígono Industrial Empresarium, Calle Romero 12, 50720 Zaragoza, Spain; ∇ TECNALIA, Basque Research and Technology Alliance (BRTA), Health Division, Parque Tecnológico de Álava, Leonardo Da Vinci, 11, E-01510 Miñano, Araba, Spain

**Keywords:** bioplastics, composites, blends, pyrolyzed lignin, electrical devices, electrodes, touch screens, EMI shielding

## Abstract

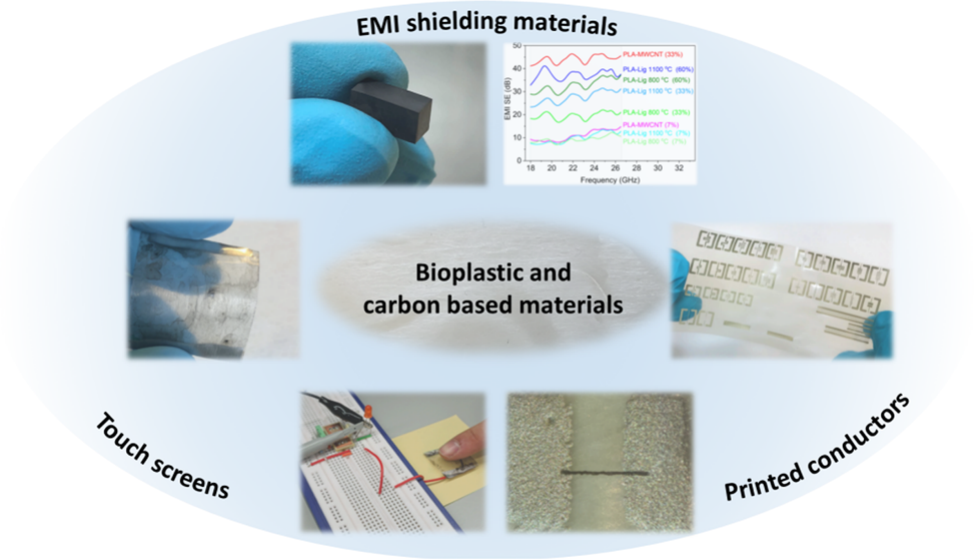

The continuously growing number of short-life electronics equipment inherently results in a massive amount of problematic waste, which poses risks of environmental pollution, endangers human health, and causes socioeconomic problems. Hence, to mitigate these negative impacts, it is our common interest to substitute conventional materials (polymers and metals) used in electronics devices with their environmentally benign renewable counterparts, wherever possible, while considering the aspects of functionality, manufacturability, and cost. To support such an effort, in this study, we explore the use of biodegradable bioplastics, such as polylactic acid (PLA), its blends with polyhydroxybutyrate (PHB) and composites with pyrolyzed lignin (PL), and multiwalled carbon nanotubes (MWCNTs), in conjunction with processes typical in the fabrication of electronics components, including plasma treatment, dip coating, inkjet and screen printing, as well as hot mixing, extrusion, and molding. We show that after a short argon plasma treatment of the surface of hot-blown PLA-PHB blend films, percolating networks of single-walled carbon nanotubes (SWCNTs) having sheet resistance well below 1 kΩ/□ can be deposited by dip coating to make electrode plates of capacitive touch sensors. We also demonstrate that the bioplastic films, as flexible dielectric substrates, are suitable for depositing conductive micropatterns of SWCNTs and Ag (1 kΩ/□ and 1 Ω/□, respectively) by means of inkjet and screen printing, with potential in printed circuit board applications. In addition, we exemplify compounded and molded composites of PLA with PL and MWCNTs as excellent candidates for electromagnetic interference shielding materials in the K-band radio frequencies (18.0–26.5 GHz) with shielding effectiveness of up to 40 and 46 dB, respectively.

## Introduction

A tremendous and continuously increasing amount of electronics waste that mankind is generating (∼54 MT in 2019) results in a massive quantity of mostly nonbiodegradable and potentially hazardous hard-to-recycle junk of polymers and metals.^1,2^ An additional problem is the growing share of fossil resources in plastic production, which is proposed to increase to 20% (from the current 4–8%) of total oil consumption by the mid of this century.^3−6^ As of today, only ∼10% of all plastics are synthesized from renewables, from which practically an insignificant fraction is applied by the electronics industry despite its overall 15% share in the use of all produced polymers.^3−8^ The dielectric substrates of printed circuit boards (PCBs) are mostly made of epoxy and phenolic resins based on fossil feedstock that leave a large CO_2_ footprint (5.7–7.6 kg CO_2_ per kg).^9^ While the currently used PCBs such as FR-4 and FR-2 show excellent electrical, mechanical, chemical, and thermal properties, there is a broad range of applications, in which mechanical flexibility and often optical transparency are necessary; thus, polyethylene terephthalate, polyether ether ketone, polyimide, and other thermoplastics are often applied. These plastics are not biodegradable either, and thus, shall be properly recycled and eventually burned at the end of their life to avoid environmental pollution and consequent adverse impacts on health.^10,11^ Furthermore, recycling of metals from the junk is also a must to avoid contamination of soil and water bodies around landfills and to save expensive critical elements including Cu, Ag, and Ni. Therefore, there are clear environmental and socioeconomic needs to introduce new polymers, blends, composites, and electrically conductive materials to the electronics industry that are based on renewables, thus supporting the concept of sustainable or *green* electronics^10−13^ prompted already in 1999.^14,15^

One way of alleviating the above problems is to substitute conventional polymers with bioplastics, as those may have end-of-life by biodegradation, do not require fossil resources, and are thus expected to have a smaller CO_2_ footprint.^3^ While the properties of currently available biobased polymers are typically inferior compared to the technical polymers developed for electronics, these materials may find applications in low-cost short-life electronics produced and used in large volumes including mostly disposable devices such as RFID tags, sensors, medical kits, and so on.^7,8,12^ Polylactic acid (PLA) is the most researched and known bioplastic that can be synthesized entirely from renewables and, depending on its use, it can be processed further to various shapes and forms by a number of different methods such as molding, extrusion, electrospinning, and three-dimensional (3D) printing.^16−18^ While PLA and its derivatives have found good use in disposable packaging and biomedical applications,^19−21^ their exploitation in electronics is less advanced because of the high thermal budgets of several classical manufacturing processes (e.g., chemical and physical vapor deposition of metals, reflow soldering of discrete components). To circumvent such limitations, lamination,^2,8,22^ low-temperature soldering,^23^ spin-coating,^24^ drop-casting,^25^ and printing (either inkjet or screen) of functional materials on the surface of the thermally sensitive PLA-based substrates have been proposed.^3,26,27^

Replacement of critical metals (e.g., Cu, Ag, Au, Pt, Pd) and indium-doped tin oxide (ITO) is another challenge. Conductive polymers (e.g., polythiophenes, polyanilines, polypyrroles) can be considered as *greener* options for some applications; however, their inferior electrical and thermal properties compared to those of metals are limiting factors.^28−30^ On the other hand, carbon-based conductive materials (such as CNTs, carbon fibers, graphene, reduced graphene oxide, pyrolytic carbon particles) are thermally more stable and have better electrical conductivities (CNTs and graphene) than those of conductive polymers. In addition, practically any known forms of potentially relevant conductive carbons may be synthesized from renewables, thus their utilization instead of metals is expected to be particularly *green*. For instance, carbon nanotubes can be grown from alcohols or oils (or from virtually any organic compounds) in the presence of a Fe, Ni, or Co catalyst.^31^ Graphene and graphene oxide may be produced by exfoliation of graphite as well as by chemical vapor deposition from methane (and also from other natural organics) on Ni and Cu surfaces,^32−34^ and pyrolytic carbon particles can be produced from abundant lignin and celluloses.^35−38^ Although the electrical conductivity of carbon-based materials is also lower than that of metals, it is sufficiently high to replace ITO in transparent conductive films (solar cells, touch panels, and sensors)^39−41^ to substitute metal electrodes (capacitors, touch sensors) and^42,43^ to find use as piezoresistive elements on polymers^44^ as well as fillers in conductive composites with polymers (electromagnetic interference shielding, piezoresistive sensors),^45−47^ which justifies and prompts their utilization in future electronics.

To promote further the concept of *green* electronics, we report on the use of bioplastics and carbon-based conductive materials that serve as environmentally benign sustainable building blocks of components and devices. We demonstrate hot-blown films of PLA-PHB blends as flexible substrates with dip-coated and inkjet-deposited carbon nanotubes for touch sensor and printed conductor applications, respectively. We also show that pyrolyzed lignin is a feasible material as electrically conductive filler in a matrix of PLA to form entirely renewable composites with highly effective electromagnetic interference shielding. In this study, we adopt several processes applied in electronics production technologies (inkjet and screen printing, dip coating, surface plasma treatment, compounding with molding) and demonstrate their compatibility with the tested bioplastics and carbon-based materials to construct functional electrical components. Accordingly, our study adds further momentum to the important and urgent exploitation of environmentally friendly materials and associated manufacturing technologies to support sustainability also in electronics.

## Results and Discussion

Flexible films of PLA-PHB and PLA-PHB-p blends were made by extrusion blowing. Depending on the processing parameters (temperature profiles in the different phases of the process, airflow rate, and/or rolling speed), the film thickness may be tuned between 30 and 140 μm. Tensile tests show anisotropy in Young’s moduli of the films with values being 2-fold in the blowing direction regardless of having plasticizer in the blend or not. On the other hand, the maximum strain at fracture is greatly improved by the addition of a plasticizer (Table 1).

**Table 1:** Mechanical Properties of Polymer Blends without (PLA-PHB) and with Plasticizer (PLA-PHB-p)^a^

sample	Young’s modulus (GPa)	yield stress at the elastic limit (MPa)	elongation at break (%)
PLA-PHB (parallel)	2.9	27	1.6
PLA-PHB (orthogonal)	1.3	26	2.1
PLA-PHB-p (parallel)	3.3	45	13
PLA-PHB-p (orthogonal)	1.5	27	14

a Terms *parallel* and *orthogonal* stand for the mechanical test direction in reference to the pull direction in the extrusion-blowing process.

According to thermal gravimetry and differential scanning calorimetry analyses (Table S1), for the PLA-PHB blend, glass transition (*T*_g_) takes place at ∼55 °C, cold crystallization (*T*_c_) at ∼120 °C, and melting (*T*_m_) at ∼152 °C. For PLA-PHB-p with a plasticizer, the *T*_g_ is at ∼55 °C as well but the cold crystallization appears at ∼102 °C and the melting point is split into ∼152 and ∼176 °C, corresponding to the melting points of PLA and PHB, respectively. The onset temperature of decomposition takes place at ∼283 °C (close to those reported for similar blends) for both types of blends,^48^ which restricts thermal budgets in postprocessing more than technical polymers used in electronics packaging.^49^

**Figure 1:**
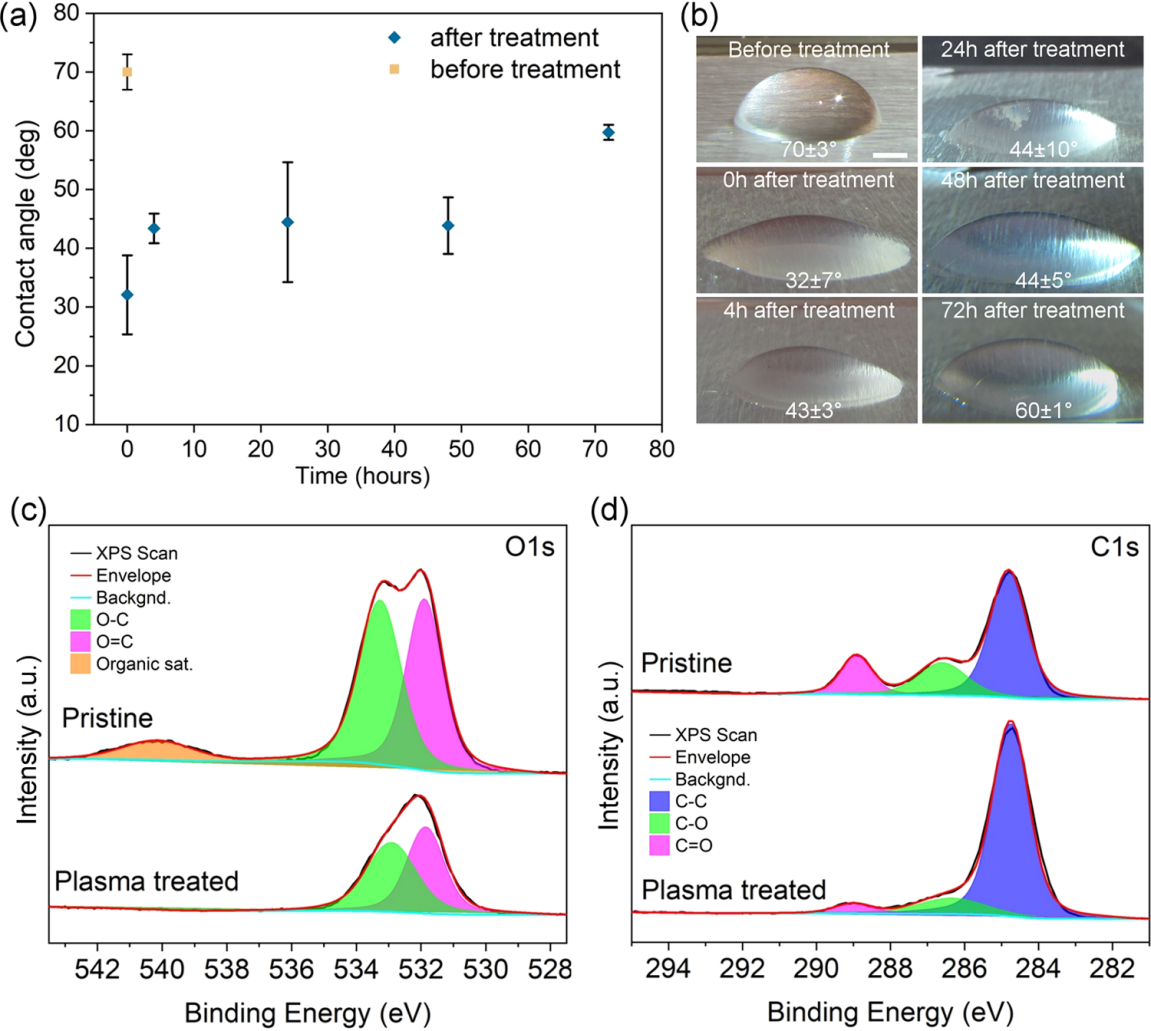
(a) Contact angle of water on the PLA-PHB surface before and after Ar plasma treatment. (b) Optical images of the water droplets with measured contact angles. Resolved X-ray photoelectron peaks (c) O 1s and (d) C 1s of the surface before and after the treatment.

The plasma treatment of the bioplastic PLA-PHB blend surface results in an increase of its surface energy, as indicated by the significantly decreased contact angle from ∼70 to ∼32° of a water droplet placed on the polymer (Figure 1a,b). Although shortly after the plasma treatment, the surface starts to relax according to the gradual increase of the contact angle, the improved wetting properties are available for over several tens of hours and make good use in subsequent surface processing with aqueous dispersions of, e.g., carbon nanotubes, as we have demonstrated it earlier for plasma-treated conventional polymers such as polyethylene terephthalate (PET) and polydimethylsiloxane (PDMS) substrates.^40,44^ The underlying cause of the improved wetting behavior of the bioplastic is due to the change in the chemical structure of the surface because of energetic bombardment with the Ar^+^ ions of the plasma. Resolved X-ray photoelectron spectra of the O 1s and C 1s peaks (Figure 1c,d, respectively) clearly show that the relative concentration of O–C bonds decreases compared to O=C and C–C bonds. Since the intensity of the C=O and C–O peaks (at 289 eV and at 286 eV) is much lower compared to the C–C peak (at ∼284.8 eV) after the plasma treatment, it seems that the polymer loses oxygen, and thus, the higher surface energy is likely to be caused by the scission of the polymer chains followed by the formation of dangling bonds with large dipole moments.

**Figure 2:**
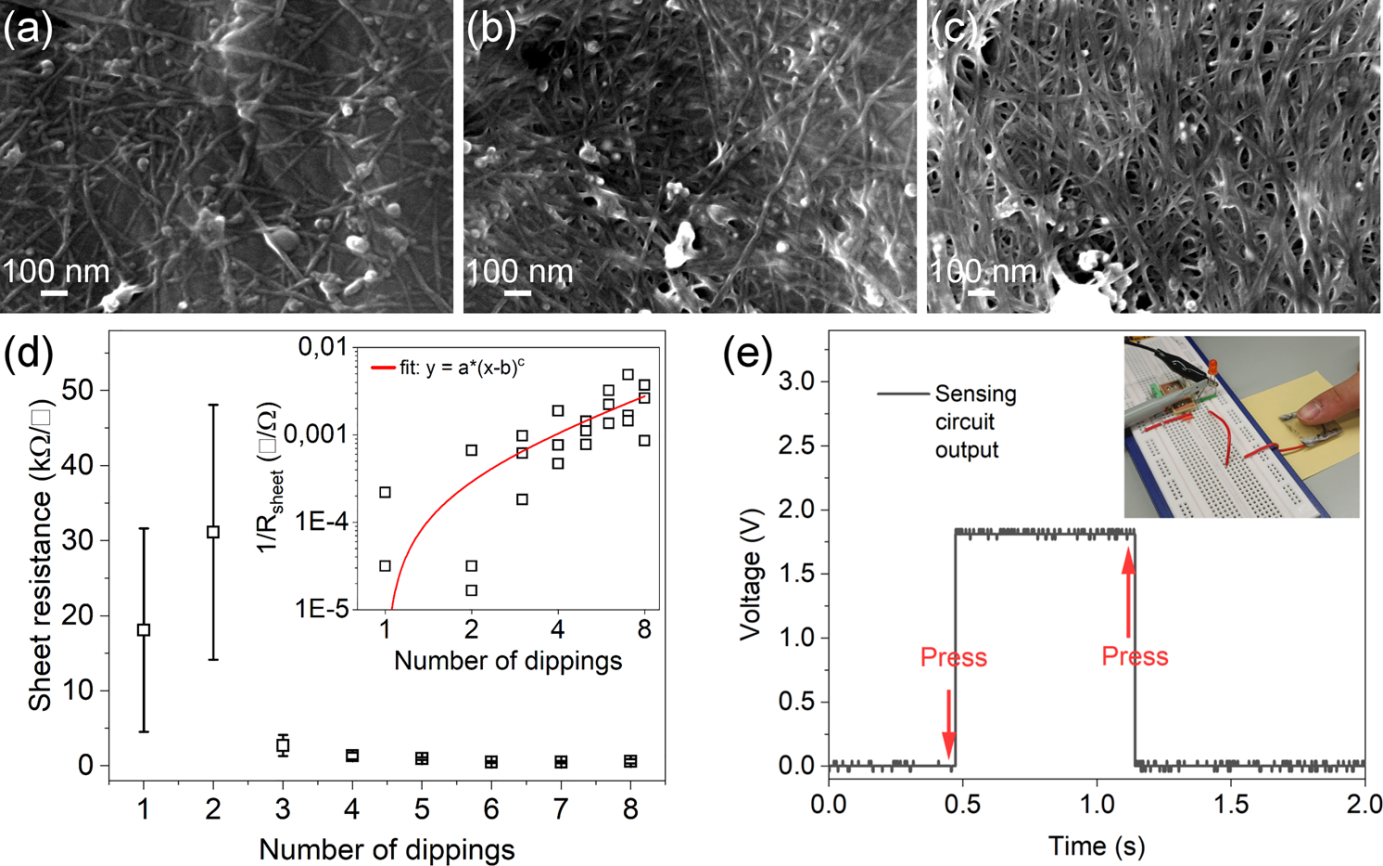
Scanning electron micrographs of plasma-treated PLA-PHB after dip coating using an aqueous dispersion of carboxyl-functionalized SWCNTs: (a) 1 dip, (b) 4 dips, and (c) 8 dips. (d) Sheet resistance versus dip coating repetition of SWCNT films deposited on PLA-PHB. Inset: log–log plot of film conductivity (the reciprocal of the measured sheet resistance) as a function of repeated dipping steps with power fit (Belehradek) having exponent *c* = 1.16 ± 1.18. (e) Capacitive touch sensor operation based on a carbon nanotube film electrode deposited on one side of the bioplastic substrate (the other capacitor plate is the finger touching the other side of the substrate). When a finger is pressing the insulating side of the film, the capacitance of the structure increases and the readout circuit makes switching of the voltage on its output. Upon repeated pressing, the capacitance is increased again and the circuit is switching the voltage on the output.

Capitalizing on the good wetting behavior of the plasma-etched surface, it is rather straightforward to deposit uniform networks of carbon nanotubes by simply dip coating the bioplastic in their aqueous dispersions. Even after a single dip coating and drying step, we find a homogenous random network of the nanotubes formed on the surface. The deposited network is percolated well, as we may conclude from the measured sheet resistance being in the range of ∼10 kΩ/□. By repeating the dipping/drying cycles multiple times, the sparse network becomes denser, more interconnects form, and thus, the conductivity of the network improves (Figure 2a–d and Supporting Information Figure S1). The trend can be described well with the percolation model *y* = *a*(*x* – *b*)*^c^*, where *y* is the conductivity and *x* is the independent variable related to the layer thickness or surface density (in our case, it is the number of performed dipping cycles). While the fitting parameter “*a*” is only a proportionality constant, parameter “*b*” shows the percolation threshold, and the exponent “*c*” of the power function describes the dimensionality of the percolation. According to the fitting on the experimental data, *c* = 1.16, which is close to the theoretical value (1.33) for percolation in 2-dimension (inset in Figure 2d), and like those found for carbon nanotube networks deposited on plasma-treated PET surfaces using the same method.^40^ The sheet resistance of the nanotube networks could be decreased well below 1 kΩ/□ after 6 dips (Figure 2d), and the as-obtained films could fulfill the function of a capacitor plate electrode as demonstrated in a touch sensor application (Figure 2e).

Printing methods as environmentally and economically benign additive manufacturing technologies to deposit functional films and micropatterns on surfaces are attractive from many aspects including, e.g., cost, speed, accuracy, and volume, thus play important roles in the production of microelectronics components and devices. Therefore, it is plausible to assess how well our bioplastics could perform as a substrate material. To test, we apply inkjet as well as screen printing methods on hot-blown PLA-PHB blend films.

**Figure 3:**
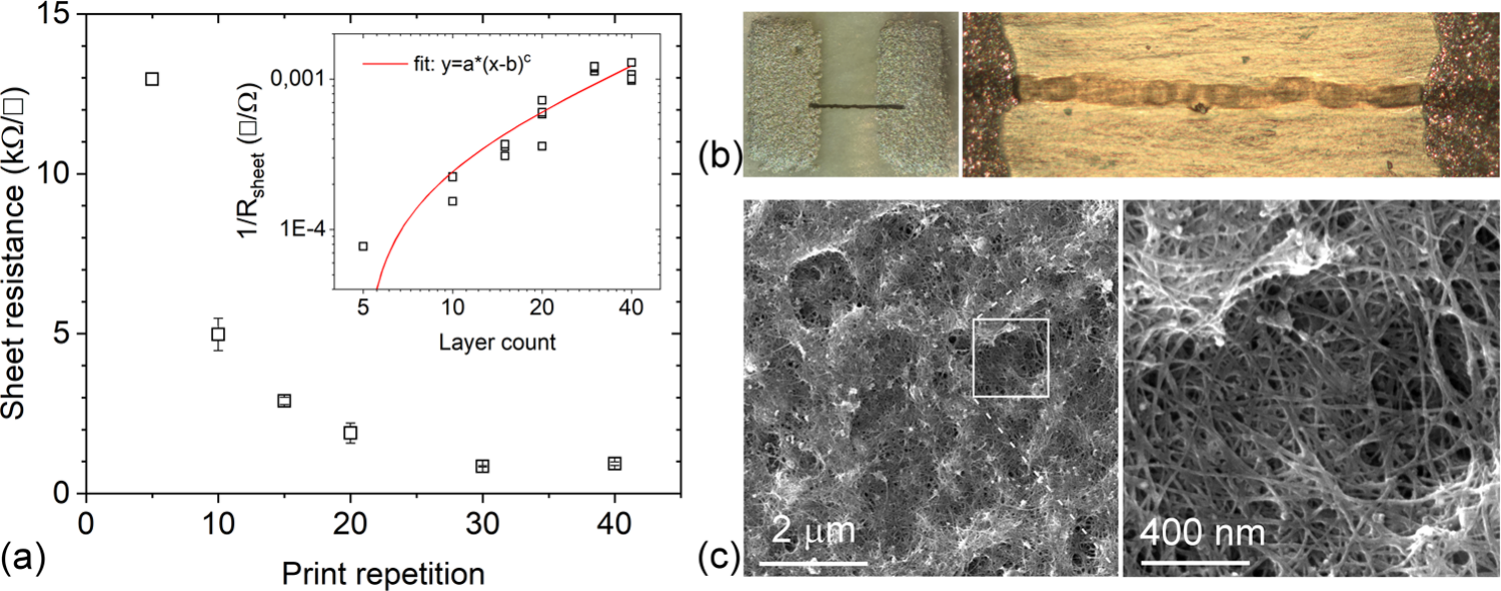
(a) Sheet resistance of inkjet-deposited SWCNT line patterns as a function of print repetition on the surface of PLA-PHB blend films. Inset: log–log plot of conductivity versus print repetition with the Belehradek percolation fit having a power exponent *c* = 0.84 ± 0.15. (b) Optical images of SWCNT line patterns inkjet-deposited between screen-printed Ag contact pads. (c) Low- and high-magnification field emission scanning electron microscopy (FESEM) images of the SWCNT network deposited with 40 print repetitions. The length and width of the SWCNT lines are 1007 ± 29 and 58 ± 6 μm, respectively.

To ensure good wetting of the surface with the aqueous carbon nanotube inks similar to those used in the dip-coating experiments, we apply Ar plasma-treated surfaces again. On such surfaces, the printed patterns of the carbon nanotubes have good line definition (average line width of 58 ± 6 μm) with moderate sheet resistance having values in the range between 10 and 1 kΩ/□ depending on the number of repeated printing scans (5 and 40, respectively) over the same pattern, which is fairly similar to those obtained with inkjet-deposited single-walled carbon nanotubes on alumina,^50^ Si/SiO_2_,^51^ glass,^52^ transparency foil,^53^ and better than on high glossy photo paper.^54^ Also, here, the conduction mechanism follows the percolation model, which may be fitted with the corresponding power function having an exponent of 0.84 (Figure 3). Accordingly, we may conclude that inkjet printing is a feasible method to produce conductive micropatterns on the bioplastic with comparable properties as on other substrate materials.

The adhesion of the nanotubes to the surface was found to be good enough to withstand sample handling, postprocessing, and various characterization procedures. Good adhesion is a consequence of at least two interactions, both having attractive characters. On the one hand, due to the plasma treatment, the wettability and hydrophilicity of the polymer are improved, thus the polar surface is capable of attracting carboxyl-functionalized SWCNTs (dipole–dipole interaction). On the other hand, we shall consider also van der Waals (vdW) attraction between the nanotubes and the surface (adhesion), and among the nanotubes in bundles (cohesion). When the deposited ink droplet dries (i.e., the solvent evaporates), capillary forces pull the nanotubes close to each other but also to the polymer surface, which then stick together after drying. The vdW binding energy between SWCNTs may be as high as =11.59 eV nm^–1^, which means that, e.g., the separation of two nanotubes having a length of 1 μm from each other requires more than 10 eV of energy, which is rather high.^55^ Now, by considering the adhesion factor (the ratio of adhesion and cohesion strength) for polymers and SWCNTs, which is around 0.8,^56^ we may assume that the vdW interaction between SWCNTs and the bioplastics is also about as strong as the cohesion between the nanotubes.

**Figure 4:**
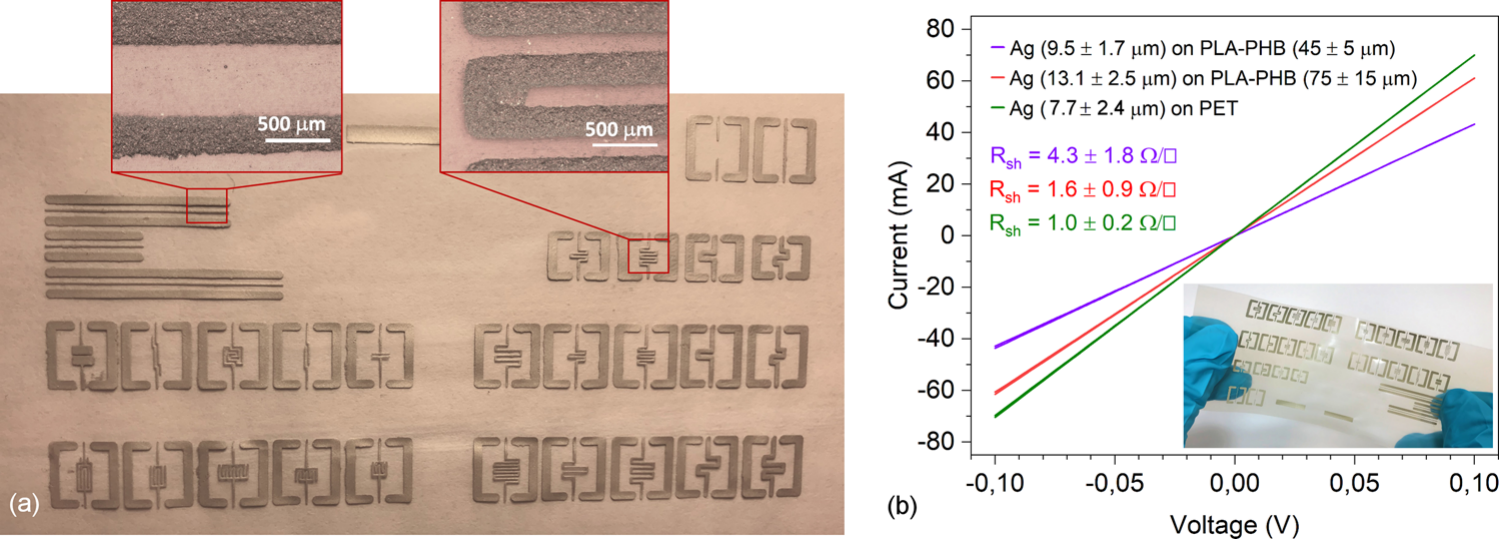
(a) Optical images of screen-printed micropatterns of Ag on the surface of a PLA-PHB-p blend film. (b) Current–voltage curves of printed Ag lines on a PLA-PHB-p of two different thicknesses as well as on a reference PET substrate.

Since the electrical conductivity of carbon-based conductor micropatterns is only sufficient for specific applications and cannot fulfill the demands put forward by conventional metal conductors of printed circuit boards, here we assess whether screen printing of metal-based inks could offer a solution to produce printouts with sufficient conductivity. In this effort, we apply a commercial Ag ink that we deposit on the blown films of PLA-PHB-p blends using manual screen printing followed by a curing step at 100 °C for 30 min. As shown in Figure 4, patterns of the Ag ink can be deposited over large surface areas without deforming or tearing the bioplastic films. In practice, the line definition is limited only by the resolution of the screen, and the quality of the printout is similar to that on a conventional PET substrate (Figure S2). However, we must note that the thermal stability (which is a known weakness of both PLA and PHB) is limiting the ultimate success of the process in terms of the electrical resistivity of the patterns. The optimum curing temperature of the ink is 130 °C (for 10–20 min), which we could not apply due to the shrinkage and breaking of the bioplastic film under such a condition. Hence, the used lower unideal curing temperature resulted in sheet resistances of 4.3 ± 1.8 Ω/□ on the thinner and 1.6 ± 0.9 Ω/□ on the thicker substrate, which is about two orders of magnitude higher than that of the technical value for the properly cured ink. Although such sheet resistance values are suboptimal when it comes to high-power and radiofrequency devices, the technology and the demonstrated materials can be still feasible to produce, e.g., contact pads (similar to those displayed in Figure 3) and short interconnects for various high-impedance circuits/devices. Anyhow, to open a broader spectrum of applications of such bioplastics in conjunction with printing methods, it is necessary to explore further techniques in the future that can produce coatings with better conductivity at very moderate temperatures, e.g., by nanotransfer^57^ and microcontact printing^58^ or by dry deposition.^59^ Another, even more versatile solution would be if the thermal stability of the polymers is improved, e.g., by better cross-linking^60^ or by optimizing the contents of various fillers and additives^48,61^ to enable postprocessing at higher temperatures.

Besides substrate applications of bioplastics, these polymers have been proven to be useful as structural or other three-dimensional parts for passive components. In particular, like other polymers, by adding electrically conductive fillers into bioplastics (e.g., by melt mixing followed by injection molding), one obtains electrically conductive composites whose properties are dependent on the concentration and on the intrinsic properties of the fillers. A plausible application of such bioplastic-based conductive composites is in electromagnetic interference (EMI) shielding for several reasons. It is known that a higher conductivity of the shielding material leads to better EMI shielding effectiveness (SE), a property that shows the power loss of propagating electromagnetic waves in a material. However, from the materials point of view, sufficiently good EMI SE values (i.e., above 30 dB) do not require electrical conductivities significantly exceeding 0.1 S/m, assuming the thickness of the shield is sufficient. Such conductivity values can be easily achieved with polymer composites. Further general advantages of the polymer-based EMI shielding materials are their low density and cost compared to metals. In addition, polymer-based materials are also relatively simple to apply as coatings on virtually any surface with arbitrary shape and size. Moreover, if the conductive filler is not metal but carbon-based, the composite is even more environmentally friendly. Accordingly, composites of bioplastics (mostly PLA) with various carbon fillers such as biochar/graphite,^46^ carbon nanotubes,^62^ carbon black,^63,64^ and graphene nanoplatelets^65^ have gained considerable momentum very lately.

**Figure 5:**
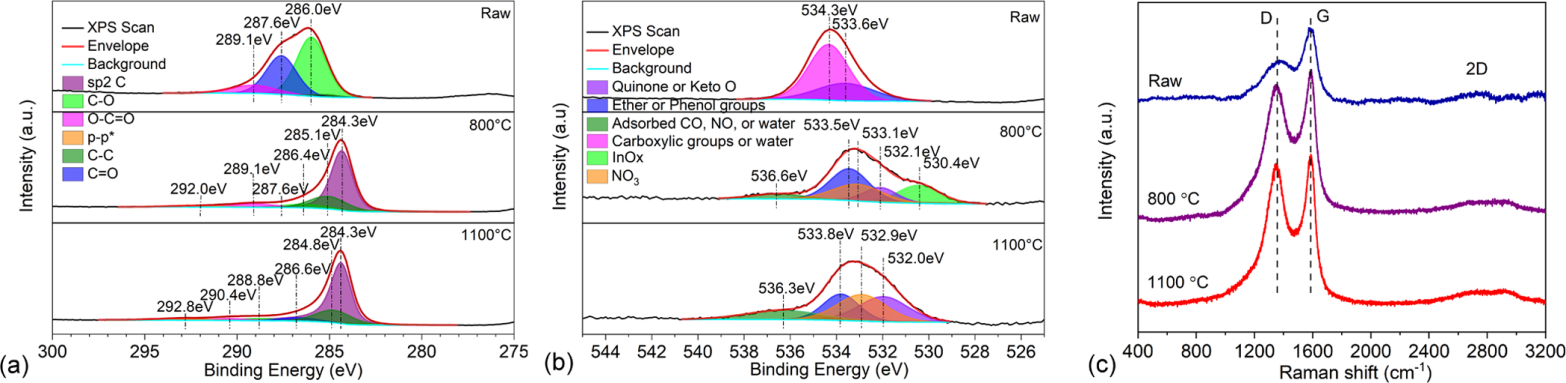
Resolved X-ray photoelectron spectra of (a) C 1s and (b) O 1s peaks, and (c) Raman spectra of untreated (raw) and pyrolyzed (800 and 1100 °C) lignin.

Here, we show a new type of conductive composite of PLA with pyrolyzed lignin (PL) as a filler. X-ray photoelectron spectroscopy (XPS) analysis of lignin and its pyrolyzed products show that carbonization of the material takes place at 800 °C. The resolved C 1s peak of the samples clearly indicates that carbonyl, carboxyl, and phenol bonds are practically vanishing, whereas aliphatic and graphitic (sp^2^) bonds form in the structure upon pyrolysis. The long tail at ∼292 eV in the C 1s peak of the pyrolyzed products is due to the appearance of the resonant structure of conjugated carbons that gives rise to the conductivity of the material (Figure 5a,b). Raman measurements complement these results. The gradual shift of the D (from 1364 to 1349 cm^–1^) and G (1584 to 1582 cm^–1^) bands, their narrowing, and the appearance of the broad 2D band at around 2800 cm^–1^ (Figures 5c and S3) also support our findings on the graphitization of carbon in the pyrolyzed samples.^66,67^

**Figure 6:**
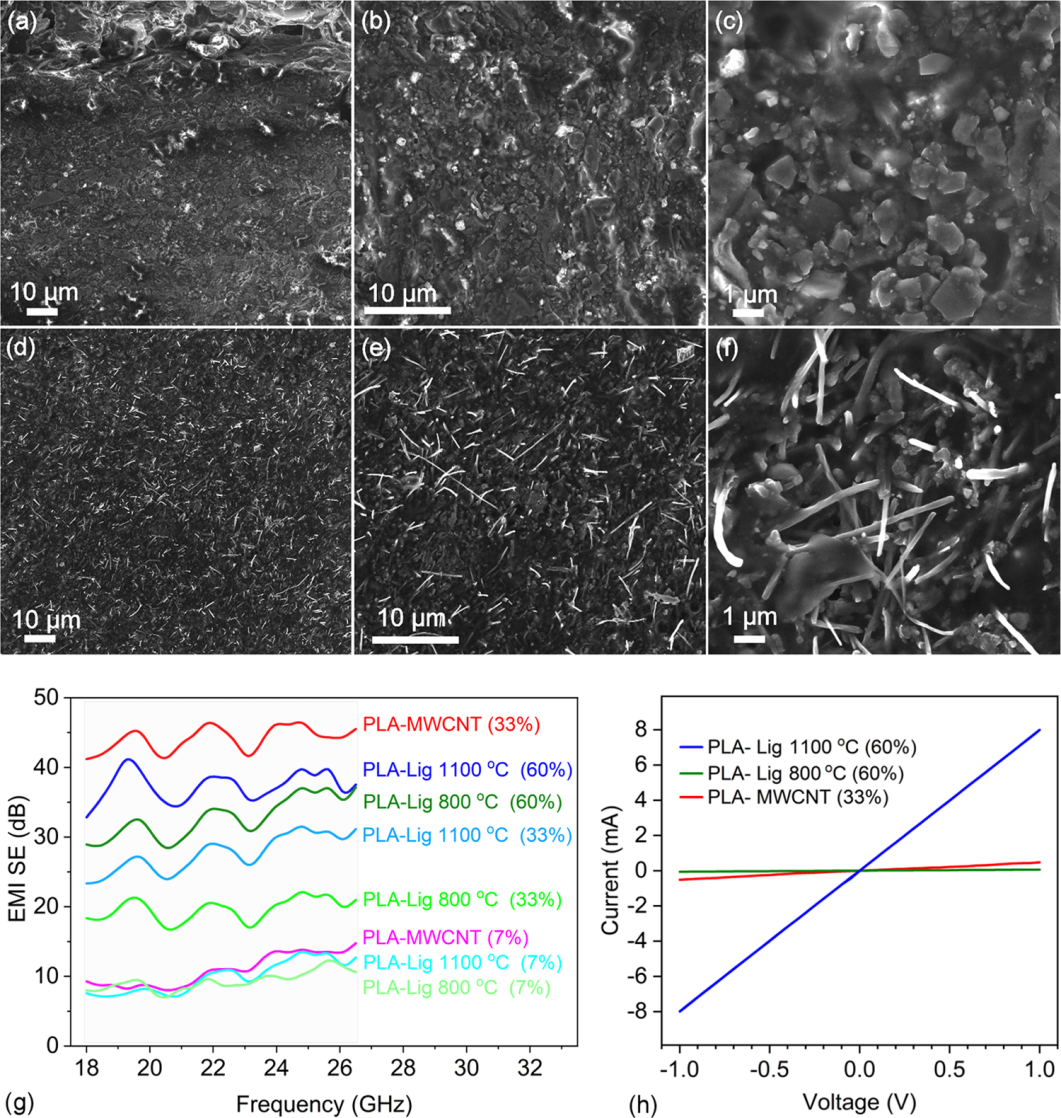
FESEM images of composites of (a–c) PLA and pyrolyzed lignin (sample PLA-Lig 1100 °C (60%)) and (d–f) PLA and MWCNTs (sample PLA-MWCNT (33%)). (g) Shielding effectiveness of composites at K-band frequencies. (h) DC current–voltage curves of the composites with the highest carbon content.

SEM assessment of cross-sectional composites shows the fillers with the highest loading (60 wt % lignin pyrolyzed at 1100 °C, and 33 wt % MWCNTs as reference) are distributed homogeneously in the PLA matrix (Figure 6a–f). It is interesting to note that the mechanical properties of PLA do not seem to change significantly upon loading with such a large fraction of fillers (Figure S4). According to tensile measurements carried out at a rate of 0.125 mm/s, Young’s moduli of the samples are 152 ± 18 MPa (PLA), 136 ± 5 MPa (PLA-Lig 1100°C (60%)), and 164 ± 14 MPa (PLA-MWCNT (33%)). The largest elongation at break was found to be ∼4% for each type of specimen. The current–voltage characteristics of the corresponding composites are linear (Figure 6h) with measured DC conductivity values of 2.3 and 0.2 S/m, respectively (Table 2). The electromagnetic interference shielding properties of PLA matrix-based composites with pyrolyzed lignin fillers (pyrolyzed at 800 and 1100 °C) were compared to MWCNT fillers. The EMI SE values were measured in the K-band frequencies (between 18 and 26.5 GHz) important for current 5G and future 6G telecommunication (Figure 6g). The composites with low filler content (7 wt %) show SE values close to 10 dB, corresponding to the moderate total power losses of ∼90%. As expected, total power losses significantly increased with the filler content. For lignin pyrolyzed at 800 °C, composites show an improvement of SE from 22 to 37 dB (at 25 GHz) as the PL filler content increases from 33 to 60 wt %. In contrast, as lignin was pyrolyzed at 1100 °C, the SE values improved from ∼31 to ∼40 dB (with a similar change in the PL filler content). For each composite, the major shielding mechanism is by absorption of the electromagnetic waves (Figure S5).

**Table 2:** Conductive Composites of Bioplastics with Carbon Fillers and Their Electrical and EMI Shielding Properties^a^

bioplastic	filler	EMI SE (dB)	frequency (GHz)	conductivity (S/m)	ref
PLA	20 vol % GR + 26 vol % BC	>40	18–26.5	1.3	(43)
PLA	0.53–1.48 vol % MWCNTs	>18	8–12.5	>1.0	(57)
PLLA/PLDA	10 wt % CB	∼27	8.2–12.4	>10	(58)
PLA/PU	30 wt % CB	∼27	8.2–12.4	∼10^–2^	(59)
PLA	10 wt % GNP	>33	8.2–12.4	∼10	(60)
PLA	60 wt % PL	>32	18–26.5	2.3	this work
PLA	33 wt % MWCNT	>40	18–26.5	0.2	this work

a GR: graphite; BC: biochar; MWCNT: multiwalled carbon nanotube; PLLA: poly(l-lactide); PLDA: poly(d-lactide); CB: carbon black; PU: polyurethane; GNP: graphene nanoplatelets; PL: lignin pyrolyzed at 1100 °C.

These results are consistent with the DC resistivity data (Table 2), i.e., pyrolysis temperature increases the electrical conductivities of the composites, hence enhancing absorption and scattering losses in the medium. Although the sample with the MWCNT filler has the highest shielding effectiveness (∼46 dB or ∼99.9975%), the best-performing PL-based composite is quite close to that (∼40 dB or 99.9900%), and its SE competes with those of other PLA and carbon-based conductive composites reported in the literature (Table 2).

## Conclusions

In our study, we aimed to assess the feasibility of PLA-PHB blends and PLA-PL composites to be used for dielectric substrates and conductive electromagnetic interference shielding media. Using contact angle measurements, we showed that the surface energy of hot-blown films of PLA-PHB blends can be increased with short Ar plasma bombardment, thus making them suitable for dip coating and inkjet printing to deposit electrically conductive (*R*_sheet_ <1 kΩ/□) carbon nanotube films and micropatterns from their aqueous dispersions. We also demonstrated screen printing as a compatible technology with the PLA-PHB blends and demonstrated large area deposition of conductive Ag micropatterns (*R*_sheet_ ∼ 1 Ω/□) on the flexible substrate. In addition, we presented compounded and molded composites of PLA with PL and MWCNTs with excellent electromagnetic interference shielding effectiveness (up to 40 and 46 dB, respectively) at microwave frequencies (18.0–26.5 GHz). The results along with the survey of contemporary literature imply that despite their inferior thermal and mechanical properties compared to those of conventional technical polymers, bioplastics may be suitable for several processes used in the manufacturing of microelectronics components and devices. Furthermore, carbon-based conductive materials could also be good alternatives to critical metals, even without trade-off, in applications that do not require ultimately high electrical conductivities (e.g., capacitive electrodes, short interconnects). Accordingly, we trust that our work along with early reports cited in the paper will motivate both the scientific community and microelectronics industry to develop further and exploit environmentally benign materials, thus contributing to a *greener* and more sustainable products in the future.

## Materials and Methods

### Dip Coating of SWCNTs on PLA-PHB and Touch-Sensing Application

The blend of PLA (75 wt %) and PHB (25 wt %) was used in the experiments (PLA, Ingeo biopolymer 2003D from NatureWorks LLC, and PHB, IamNATURE B6H N15 from Gruppo MAIP). The blending of the components was carried out using a co-rotating twin-screw extruder (Leistritz 27E) in a temperature range of 165–175 °C and rotation speed of 300 rpm. The produced pellets were then melted and hot-blown using a Eurotech Extrusion Machinery device at a process condition temperature profile of 170 °C, an output of 3.5 kg/h, and a rotation speed of 35 rpm. The thickness of the as-made films was in the range of 55 ± 15 μm.

An aqueous dispersion of carboxylic acid-functionalized single-walled carbon nanotubes (SWCNT-COOHs, Sigma-Aldrich 652490) was prepared by adding 142 mg of SWCNT-COOH to 160 mL of deionized water and stirred on a magnetic plate for 30 min while adding 4 mL of NH_4_OH. After stirring for 30 min, the dispersion was sonicated for 2 h (Finnsonic M12 200W/800W), then centrifuged (Hettich Zentrifugen universal 320) for 30 min at 3000 rpm, and the supernatant was collected. The concentration of the dispersion was found to be 0.54 ± 0.07 mg/mL.

Ar plasma treatment of the bioplastic blend film was carried out using an Oxford PlasmaLab Plus facility (20 sccm flow of Ar, plasma power of 100 W, a pressure of 20 mTorr for 5 min for contact angle measurements and for 13 min for the dip-coating experiments). Immediately after the plasma treatment, the samples were subjected to the dip-coating process. In a typical experiment, the sheets were manually immersed in the aqueous nanotube dispersion, then slowly drawn out, and finally, dried in a box furnace at 40 °C for 5 min to deposit. The procedure was repeated multiple times to increase the surface coverage of carbon nanotubes.

The contact angle of water droplets (30 μL) dispensed on the surface was measured by analyzing optical images (FijiJava software). Measurements were carried out before and after the plasma treatment (0 h, 4 h, 1 day, 2 days, 3 days).

For the electrical measurements of the coated films, Pt electrodes having a thickness of ∼225 nm were sputtered on the edges of the layers through a shadow mask. The sheet resistance and conductivity were calculated from the measured sample geometry and current–voltage curves (using a Keithley 2636 source meter in a 2-probe configuration).

**Scheme 1:**
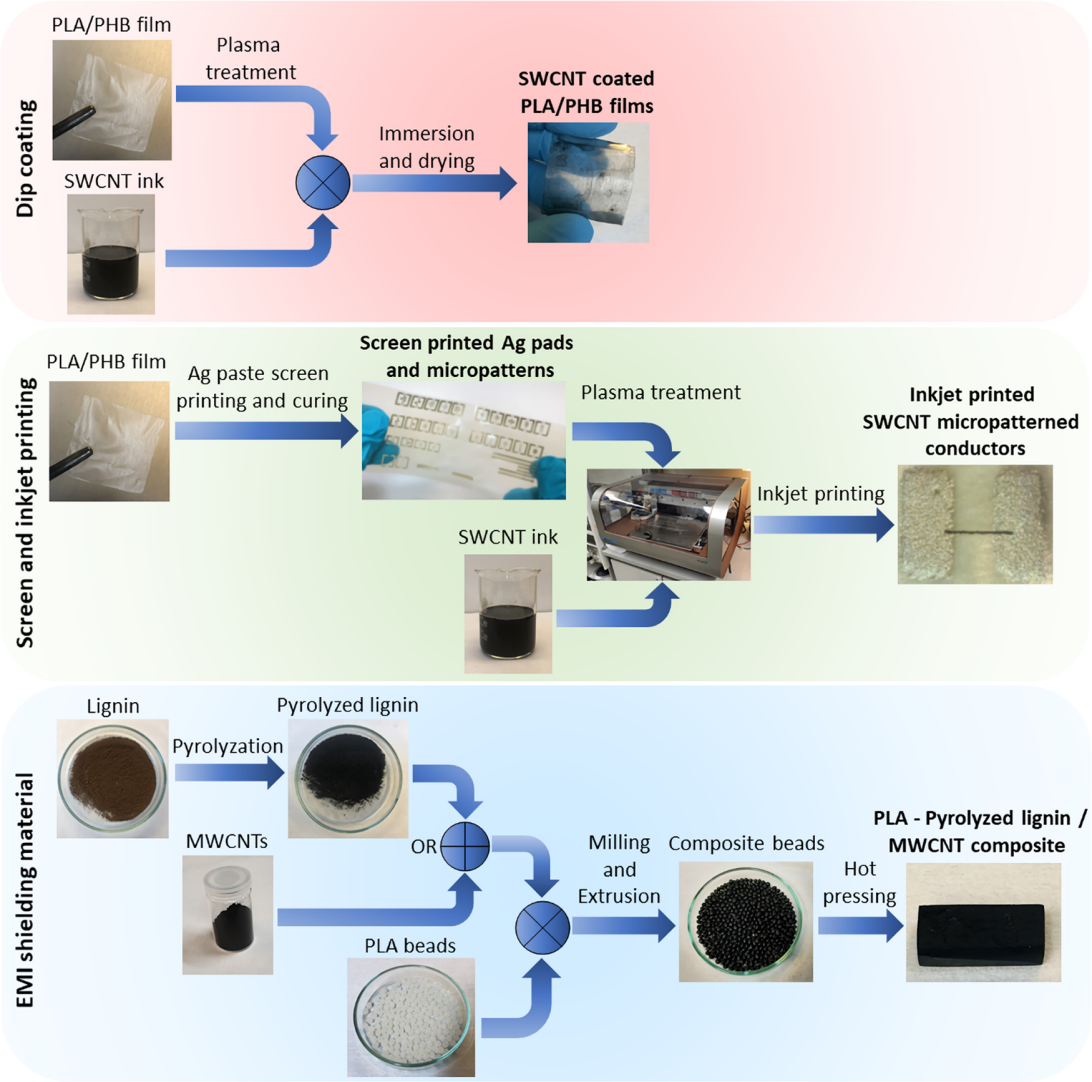
Illustration of the Methodology Used in the Processing of Materials to Prepare Electrical Components

The dip-coated sample (2 dips) was applied as a capacitor plate to demonstrate an application for touch sensing. When a finger approaches the uncoated side of the bioplastic sheet, a capacitor structure forms and is detected with a circuit (similar to that shown in ref (29)) whose output is measured with an Agilent DSO-X 3024A oscilloscope (Scheme 1).

### Inkjet Printing of Micropatterned Conductors of SWCNTs on PLA-PHB

Inkjet printing of carbon nanotubes was carried out on the same type of PLA-PHB bland bioplastic films as for the dip-coated samples. The plasma treatment parameters were also the same, except for the power used (200 W). The carbon nanotube dispersions were different in the experiments and were made by mixing 17.4 g of deionized water, 17.2 g of dimethylformamide (DMF), and 16.97 mg of SWCNT-COOH (Sigma-Aldrich 652490) for 2 min in a magnetic mixer, followed by ultrasonic dispersion (Finnsonic M12 200W/800W) for 30 min at 50 °C. The dispersion was then centrifuged (Hettich Zentrifugen Universal 320) at 3500 rpm for 10 min, and the supernatant was collected. After repeating the centrifugation and collection steps 4 times, a stable SWCNT ink (with a concentration of 0.25 mg/mL) suitable for inkjet printing was obtained.

Inkjet printing of line patterns of the nanotubes (using a Dimatix DMP-2850 materials printer) was carried out immediately after the plasma treatment of the bioplastic substrates having previously screen-printed Ag electrodes on them (electrode spacing of 1.0 mm, Ag paste Dupont 5064H). To achieve the best consistency between prints and to be able to reliably scale the manufacturing process further, when printing, the most concentrated first droplet of every horizontal pass was discarded (Scheme 1).

*I*–*V* and resistance measurements (2-probe configuration) were carried out using a Keithley 2636A sourcemeter with an automated LabVIEW script. Sweeping voltage from −2 to +2 V with a 0.1 V step and 100 ms delay in between the samples was applied with a current limit of 1 mA.

### Screen Printing of Ag Micropattern on PLA-PHB-p

The PLA-PHB-p blend used in the experiments is similar to the one described previously, except that the PHB was produced by the Bio Base Europe Pilot Plant (from potato peels as a feedstock) and 4 phr plasticizer was added (GLYPLAST OLA 2, by Condensia Química). Blending and film blowing were similar to those described earlier. The PLA-PHB biobased films used for substrates were produced in film-blowing equipment (Labtech LBM-125, vertical machine direction stretching unit) where the thermal profiles and processing parameters were adjusted accordingly to the material properties such as melting and/or degradation temperatures. Films with two different thickness values (45 ± 5 and 75 ± 15 μm) were used in the screen-printing experiments.

The printing was done by manually squeezing a low curing temperature Ag paste (DuPont 5064H) through a 67T polyester screen (Scheme 1). After printing, the samples were cured at 100 °C for 30 min. PET sheets were used as reference samples. The prints were inspected using optical microscopy (Olympus BX51) and profilometry (Bruker DektakXT). Sheet resistances and conductivities of the printed Ag patterns were calculated from the measured sample geometry and 2-point probe current–voltage data (Keithley 2636A source meter).

### PLA-Lignin and PLA-MWCNT Composites and Their EMI Shielding Analysis

Polylactic acid beads (Luminy LX175, Total Corbion), multiwalled carbon nanotubes (MWCNTs, Sigma-Aldrich, 659258), and lignin powder (St1 Cellunolix made from pine and spruce softwood by enzymatic hydrolysis, followed by filtration of the residue termed as hydrolysis lignin) were used in the experiments. The hydrolyzed lignin powder was pyrolyzed in a tube furnace at 800 or 1100 °C for 20 h in Ar atmosphere fed with a flow rate of 20 mL/min. After cooling in continued Ar flow, the pyrolysis product was milled into smaller pieces (with a broad size distribution spanning between ∼50 nm and ∼50 μm, Figure S6) in a mortar and used for subsequent compounding with PLA.

The PLA granules/pyrolyzed lignin batches with various ratios were compounded at 210 °C using a twin-screw extruder (HAAKE Minilab Rheomex CTW5, at 35 rpm for 30 min), and the extruded composite granules were then inserted in a hot press (190 °C) to produce specimens having a size of ∼10.7 × 4.3 × 5 mm^3^. The same processes were used for preparing the MWCNT and PLA composites (Scheme 1). Note that the specimens were polished to a thickness of 3.3 mm for analyzing the electromagnetic interference (EMI) shielding properties and to 2.0 mm for mechanical tensile measurements.

The microstructure and composition of the treated lignin were characterized by Raman spectroscopy (Horiba Jobin-Yvon LabRAM HR800 UV–vis μ-Raman, λ = 488 nm), field emission scanning electron microscopy (FESEM, Zeiss ULTRA plus, 15 kV), and X-ray photoelectron spectroscopy (XPS, SPECS instrument with Al Kα source operated at 150 W and 14 kV). Note that the powder samples were pressed onto indium foil for mounting.

The *I*–*V* curves of the composites were measured using a Keithley 2636A Sourcemeter in a two-probe configuration. The electromagnetic interference (EMI) shielding measurements in the K-band frequency range (18–26.5 GHz) were carried out using a Keysight WR60 rectangular waveguide with a sample holder and 2-port Agilent 8517B S-parameter Test Set. The waveguide measurement setup was calibrated with the standard two-port method in forward and reverse direction by measuring through, short and load. The EMI shielding effectiveness (SE) values and its reflection (SE_R_) and absorption (SE_A_) components were calculated from the measured *S*_11_ and *S*_21_ scattering parameters as described elsewhere:^68^ SE = SE_R_ + SE_A_, where SE_R_ (dB) = 10 log[1/(1 – |*S*_11_|^2^)] and SE_A_ (dB) = 10 log[(1 – |*S*_11_|^2^)/|*S*_21_|^2^]. Note that SE(%) = (1–10^–0.1SE^)*100%.

## Supporting Information Available

Thermal properties of the bioplastic blends; low-magnification scanning electron micrographs of plasma-treated PLA-PHB after dip coating; optical images of screen-printed micropatterns of Ag on the PET surface; resolved Raman spectra of lignin before and after pyrolysis at different temperatures; mechanical stress–strain curves of PLA and its composites with pyrolyzed lignin and carbon nanotubes; contribution of reflection (SE_R_) and absorption (SE_A_) losses to the total shielding effectiveness (SE) for different materials at 25 GHz; SEM images of lignin before and after pyrolysis at different temperatures (PDF)
