# Supplementary material for: Bioplastics and Carbon-Based Sustainable Materials, Components, and Devices: Toward Green Electronics
Source: ACS Appl Mater Interfaces. 2021 Oct 5;13(41):49301–12. doi: 10.1021/acsami.1c13787 (PMC8532127; doi:10.1021/acsami.1c13787)
Supplement: Supplementary file 1 — am1c13787_si_001.pdf [file am1c13787_si_001.pdf]

Supporting information

# Bioplastics and carbon based sustainable materials, components and devices: Towards *green* electronics

Éva Bozó,[1] Henri Ervasti,[1] Niina Halonen,[1,*] Seyed Hossein Hosseini Shokouh,[1] Jarkko Tolvanen,[1] Olli Pitkänen,[1] Topias Järvinen,[1] Petra S. Pálvölgyi,[1] Ákos Szamosvölgyi,[2,3] András Sápi, [2,3] Zoltan Konya,[2,3] Marta Zaccone,[4] Luana Montalbano,[4] Laurens De Brauwer,[5] Rakesh Nair,[5] Vanesa Martínez-Nogués,[6] Leire San Vicente Laurent,[7] Thomas Dietrich,[7] Laura Fernández de Castro,[7] Krisztian Kordas[1,*]

[1] *Microelectronics Research Unit, Faculty of Information Technology and Electrical Engineering, University of Oulu, PO Box 4500, FI-90570 Oulu, Finland*

[2] *Department of Applied and Environmental Chemistry, University of Szeged, Rerrich B. tér 1, Szeged 6720, Hungary*

[3] *MTA-SZTE Reaction Kinetics and Surface Chemistry Research Group, University of Szeged, Rerrich B. tér 1, Szeged 6720, Hungary*

[4] *Proplast - Consorzio per la Promozione della Cultura Plastica, Via Roberto di Ferro, 86 - 15122 Alessandria, Italy*

[5] *Bio Base Europe Pilot Plant VZW, Rodenhuizekaai 1, 9042 Desteldonk (Gent), Belgium*

[6] *Tecnopackaging, Polígono Industrial Empresarium, Calle Romero 12, 50720, Zaragoza, Spain*

[7] *TECNALIA, Basque Research and Technology Alliance (BRTA), Health Division, Parque Tecnológico de Álava, Leonardo Da Vinci, 11, E-01510 Miñano – Araba, Spain*

* Authors to whom correspondence shall be addressed: niina.halonen@oulu.fi and krisztian.kordas@oulu.fi

**Table S1** Thermal properties of the bioplastic blends used in the experiments. $T_g$, $T_c$, $T_m$ and $T_{onset}$ are the temperatures of glass transition, cold crystallization, melting and the start of degradation, respectively.

| **Sample** | **$T_g$ (°C)** | **$T_c$ (°C)** | **$T_m$ (°C)** | **$T_{onset}$ (°C)** | |
|---|---|---|---|---|---|
| | | | | $N_2$ | Air |
| PLA-PHB | 56 | 121 | 151 | 282 | 284 |
| PLA-PHB-p | 55 | 103 | 152, 176 | 281 | 283 |

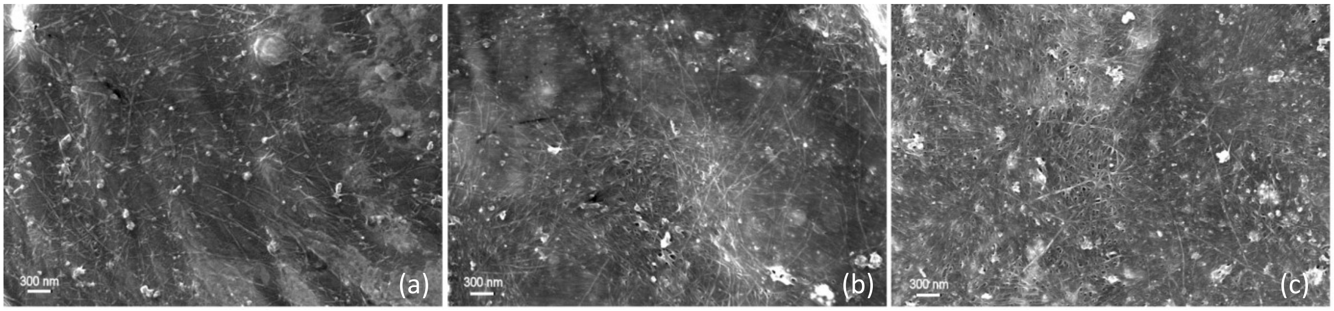


**Figure S1** Low magnification Scanning electron micrographs of plasma treated PLA-PHB after dip coating using aqueous dispersion of carboxyl functionalized SWCNTs: (a) 1 dip, (b) 4 dips and (c) 8 dips.

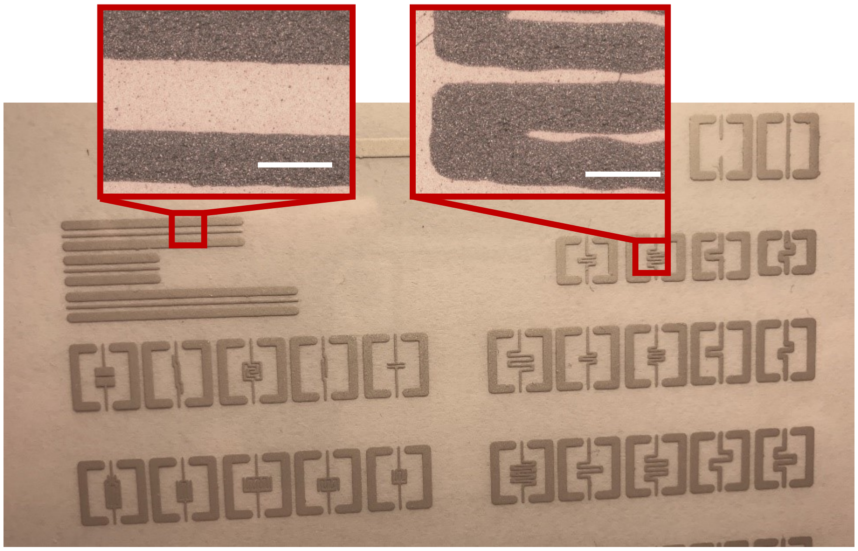

**Figure S2** Optical images of screen printed micropatterns of Ag on the surface of PET. Scale bars: 500 µm.

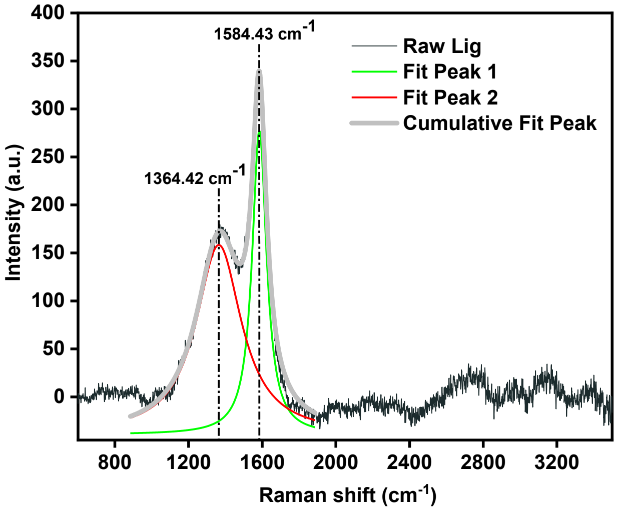


(a)

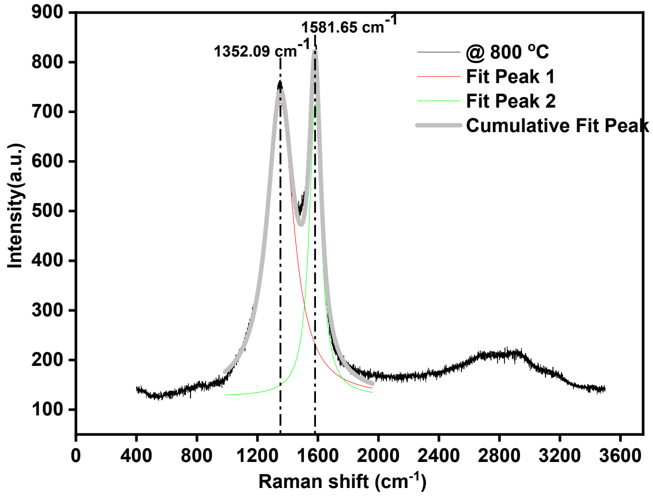


(b)

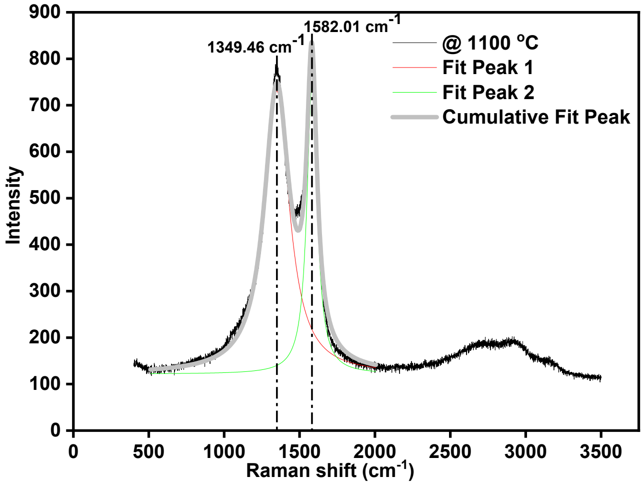


(c)

**Figure S3** Resolved Raman spectra of lignin before and after pyrolysis at different temperatures. (a) Pristine, (b) pyrolyzed at 800°C and (c) pyrolyzed at 1100°C.

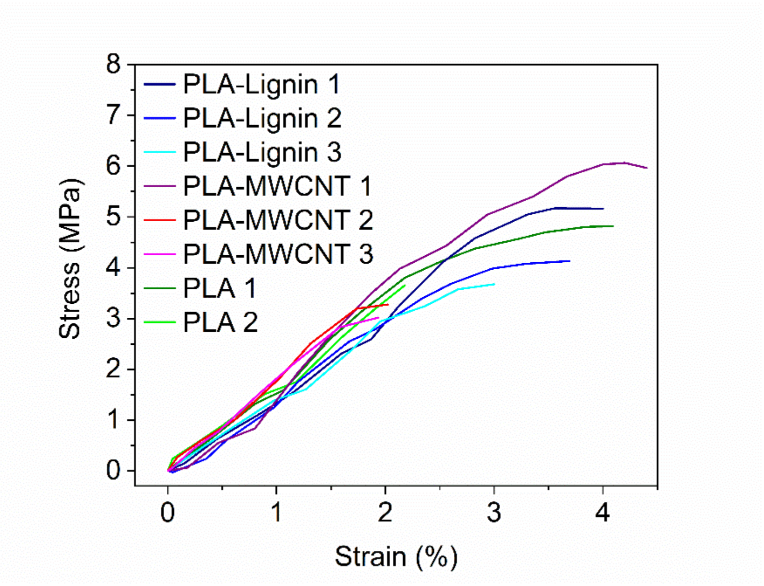


**Figure S4** Stress-strain curves of the molded PLA as well as it composites with pyrolyzed lignin or carbon nanotubes. In the legend of figure, 1, 2 and 3 denote specimens produced in different experimental batches. Note: Because of the inaccuracy of the starting point registration of the experimental setup, the zero point offset of strain is manually corrected in the displayed plot.

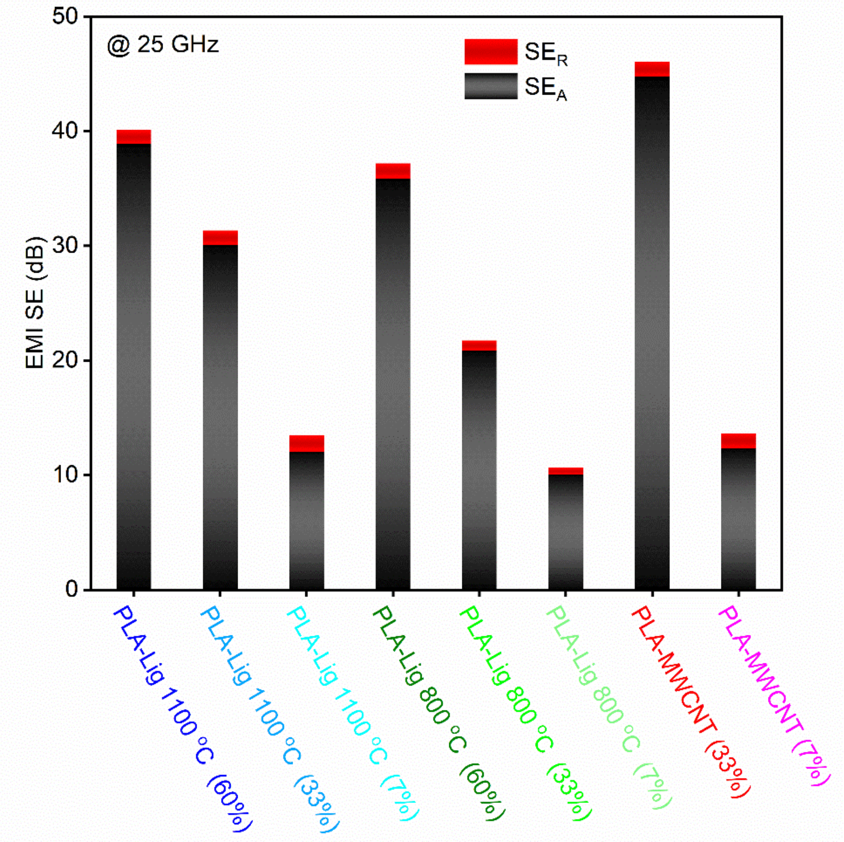


**Figure S5** Contribution of reflection (SE$_R$) and absorption (SE$_A$) losses to the total shielding effectiveness (SE) for different materials at 25 GHz.

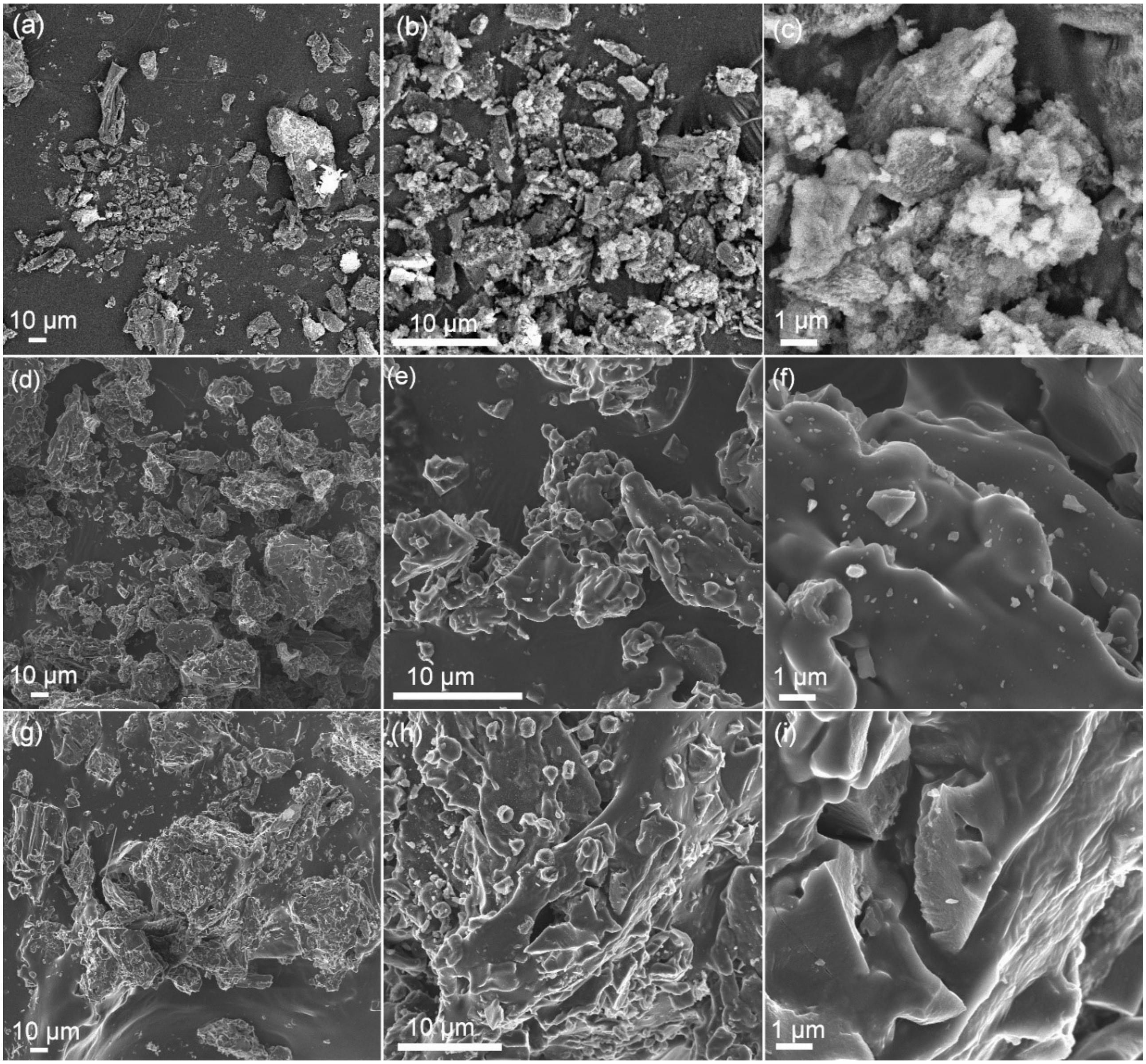


**Figure S6** SEM images of lignin before and after pyrolysis at different temperatures. (a-c) Pristine, (d-f) pyrolyzed at 800°C and (g-i) pyrolyzed at 1100°C.
